# Factors Associated With Anemia Among Orang Asli Children Aged Two to Six Years in Negeri Sembilan, Malaysia

**DOI:** 10.7759/cureus.35511

**Published:** 2023-02-26

**Authors:** Siti Fatihah Murtaza, Ling Jun Lee, Nur Nadhirah Usaini, Wan Ying Gan, Norhasmah Sulaiman

**Affiliations:** 1 Department of Nutrition, Universiti Putra Malaysia, Serdang, MYS

**Keywords:** anemia, parasitic infections, food security, malaysia, orang asli children, anaemia

## Abstract

Background

Anemia is a global public health problem that needs urgent attention, especially in early childhood. Young children living in remote indigenous communities are vulnerable to anemia. This study aimed to determine factors associated with anemia among children of the Orang Asli (OA) community, aged two to six years old.

Methods

A cross-sectional study was conducted among 269 OA children, together with their biological non-pregnant mothers. Their mothers were interviewed using a structured questionnaire to gather information on sociodemographic characteristics, sanitation facility and personal hygiene, food security, and dietary diversity. Anthropometric and biochemical assessments were measured using standard protocols.

Results

One in five of the OA children was anemic (21.2%) and had a low birth weight (20.4%). About 27.7% of the children were underweight, 35.2 % were stunted, 6.1% were wasted, and 5.7% were overweight. One-third of them (35.0%) had parasitic infections and almost all were food-insecure (96.3%). As for the mothers, more than one-third of them were anemic (39.0%), 58.9% had abdominal obesity, and 61.8% were overweight and obese. Parasitic infections (adjusted OR (AOR)=2.49, 95%CI=1.23-5.06), not wearing shoes outside the house (AOR=2.95, 95%CI=1.39-6.27), and mothers with anemia (AOR=2.62, 95%CI=1.30-5.28) were associated with increased risk of anemia among OA children.

Conclusion

Preventing maternal anemia and strengthening knowledge on sanitation and hygiene could be incorporated into nutrition intervention programs to address anemia issues among OA children.

## Introduction

Anemia is a serious global public health problem that warrants urgent attention. It is a condition in which the number of erythrocytes or the concentration of hemoglobin (Hb) falls below average and becomes inadequate to meet the physiological needs of an individual [1]. Anemia is defined by the World Health Organization (WHO) as having a Hb concentration below 11.0 g/dL in children aged 6-59 months [1]. Globally, the prevalence of anemia at all ages was 22.8% (95 CI: 22.6-23.1) in 2019 [2]. Of these, 39.7% of children under five years old were anemic [2]. Besides being one of the leading age groups to be diagnosed with anemia, this age range also represents a crucial time for the growth and development of children [2].

Childhood anemia adversely affects the short- and long-term emotional, physical, and social development of children. It can also trigger immune function defects, poor motor and cognitive growth, poor educational performance, and decreased productivity in children’s lives, thus diminishing the income capacity and adversely affecting national economic growth in the long term [1-3]. As it is a critical issue, more studies related to anemia among preschool children should be conducted globally, including in Malaysia.

Worldwide, indigenous children have been associated with poorer health outcomes compared to non-indigenous children [4]. Similarly, studies have reported a disproportionate burden of anemia among indigenous people compared to non-indigenous populations globally [5,6]. Indigenous people of Peninsular Malaysia (e.g., the Senoi, Proto Malay, and Negrito tribes) are collectively known as “Orang Asli (OA)”, i.e., the original people in Malay. As of 2019, the OA were estimated to account for about 14% of the population of Malaysia [7]. Anemia is a significant public health issue among the OA population, with a prevalence ranging from 25.9% [8] to as high as 41.5% [9] among OA children. Thus, it is vital to investigate the prevalence of anemia among OA children and its associated factors.

Anemia in children is associated with multifactorial factors from environmental, household, and nutritional aspects [10-12]. Previous studies have reported a variety of risk factors for anemia, including sociodemographic factors (family income, number of children, mother’s educational level), nutritional status (child’s stunting and dietary diversity, and maternal anemia status) [10,11,13]; as well as poor sanitation and hygiene [11,14]. However, limited studies have been conducted to assess factors associated with anemia among OA preschool children [8,9].

Given the importance of the crucial period of childhood to prevent negative impacts of anemia, it is important to investigate the risk factors of anemia in early childhood, especially among OA children who are vulnerable. Therefore, this study aimed to determine factors associated with anemia among OA children aged two to six years old. This study would be beneficial for nutrition intervention programs to address anemia problems among OA children.

## Materials and methods

Study design and participants

A cross-sectional study was conducted at the OA villages in Negeri Sembilan, Malaysia, between April 2015 and January 2016. Two districts, namely Kuala Pilah (14 villages) and Jempol (16 villages), were purposely selected due to the high number of OA villages. Of these, only 14 OA villages (eight from Kuala Pilah district and six from Jempol district) agreed to participate in this study. All OA children aged two to six years old, together with their biological non-pregnant mothers were approached (n=280), with an overall response rate of 96.1% (n=269). OA children who were suffering from chronic illness, neurological deficit, Down’s syndrome, cerebral palsy, mental retardation, severe food allergies, or any condition that might affect appetite, feeding, or growth were excluded from this study. The sample size of this study was calculated by comparing the proportions of the prevalence of anemia among underweight (56.4%) and non-underweight (32.4%) children [15], with an α-value of 0.05 and 80% power. The required minimum sample size was 160 after considering a 20% non-response rate and a design effect of two.

Ethical approval was obtained from the Ethics Committee for Research Involving Humans Subjects, Universiti Putra Malaysia (JKEUPM), Serdang, Malaysia (approval number: FPSK (FR15) P001). This study was conducted with the approval of the Department of Orang Asli Development (JAKOA). Written informed consent was obtained from all the mothers prior to data collection.

Data collection

Three visits were conducted in the selected OA villages. Mothers were interviewed using a structured questionnaire and 24-hour diet recall during the first home visit. The second home visit was conducted to interview mothers on the second day of the 24-hour diet recall and to collect the stool samples of the children. On the third visit, all participants (children and their mothers) were gathered in the village hall to obtain their blood samples and anthropometry measurements.

Measures

Sociodemographic Characteristics

Information on the child’s age, sex, birth order, birth weight, parental age, education level, employment status, number of family members, and monthly household income were obtained from mothers.

Sanitation and Hygiene

The questions on personal hygiene, household environment, and sanitation facilities were adapted from a past study [16]. Observations were made to assess the personal hygiene of the children, household environment, and sanitation facilities. A binary option of yes/no was used.

Food Insecurity

The 10-item Radimer/Cornell Hunger and Food Insecurity Instrument [17] was used to measure the food insecurity of the participants. The response options included ‘not true’, ‘sometimes true’, and ‘often true’. Each participant would then be classified by the severity of food insecurity, namely food security, household insecurity, individual insecurity, or child hunger. In this study, Cronbach’s alpha coefficient was 0.86, indicating good internal consistency reliability.

Dietary Diversity

The dietary diversity of the children was measured using a dietary diversity questionnaire based on the average score from the two-day 24-hour dietary recall [18]. Twelve food groups considered were cereals, white tubers and roots, vegetables, fruits, meat, eggs, fish and other seafood, legumes, nuts and seeds, milk and milk products, oils and fats, sweets, spices, condiments, and beverages [18]. The intake of each food group by the child would be given a one-point score based on the two-day 24-hour dietary recall. In contrast, a zero point was given if the food in the particular group was not consumed. The dietary diversity score (DDS) was generated by summing up the scores from each food group. A high DDS reflects a more diverse diet with more food groups being consumed.

Anthropometric Measurements

The birth weight of the children was collected from their health record books. The current height and body weight of the mothers and children were measured with a seca portable stadiometer 213 (seca GmbH & Co., Hamburg, Germany) to the nearest 0.1cm, and a TANITA Digital Weight Scale THD-306 (TANITA Corporation, Tokyo Japan) to the nearest 0.1kg, respectively. Mothers with a height of less than 145cm were classified as having short stature [19]. The body weight status was determined according to the WHO BMI classifications for adults. The waist circumference of the mothers was measured with a seca 201 measuring tape (seca GmbH & Co.) to the nearest 0.1cm. Mothers with a waist circumference of 80cm and above were classified as having abdominal obesity. The mean z-scores for weight-for-age (WAZ), height-for-age (HAZ), and BMI-for-age (BAZ) of the children were generated according to the WHO Child Growth Standard 2006 among children under five years old and the WHO Growth Reference 2007 among children aged five and above.

Hb Concentration

Blood sampling was carried out by a pediatrician via the finger prick method. Hb concentrations of the children and mothers were measured using a digital blood hemoglobin measurement meter, HemoCue Hb 201+ Analyzer (HemoCue AB, Angelholm, Sweden). The Hb levels of the children and mothers were determined according to the classification of anemia [1]. Non-pregnant women with Hb concentration less than 12g/dL, children aged 6-59 months with Hb concentration less than 11g/dL, and children aged five to six years with Hb concentration less than 11.5g/dL were classified as having anemia [1].

Parasitic Infection

A thumb-sized fresh stool sample from the children was collected by their mothers. The stool sample must be free of urine, water, or sand and placed in the stool container provided. Once the stool was collected, mothers contacted the researcher to collect the stool sample from their house to be transported in an icebox to an accredited laboratory for further analysis. Stool samples were analyzed to identify the existence of intestinal parasites, namely *Trichuris trichiuria* and *Ascaris lumbricoides.*

Statistical analysis

Data analysis was performed by using IBM SPSS Statistics for Windows, Version 27.0 (2020; IBM Corp., Armonk, New York, United States). Skewness was set within the range of -2 to +2 to determine the normal distribution of data [20]. Descriptive statistics were used to describe the distribution of data in the form of mean and standard deviation for continuous variables as well as frequencies and percentages for categorical variables. Chi-square (χ^2^) test and Fisher exact test were used to determine the associations between categorical variables and the anemia status of the children (anemic and non-anemic groups). All variables with a p<0.25 in the Chi-square test were included in the multiple logistic regression model to determine factors associated with anemia among OA children. The statistical significance level was set at p<0.05.

## Results

A total of 269 OA children (50.9% males and 49.1% females) participated in this study (Table 1). The prevalence of anemia among OA children in this study was 21.2%. Results showed that birth weight (χ2=3.91, p=0.048) and father's education level (χ2 = 10.04, p=0.018) were associated significantly with anemia status of the children.

**Table 1:** Sociodemographic characteristics of the OA children and parents (n = 269). *significant at p<0.05

Characteristics	n (%)			χ^2^	p-value
	Non-anemic (n = 212)	Anemic (n = 57)	Total (n = 269)		
Children					
Sex				0.08	0.772
Male	107 (78.1)	30 (21.9)	137 (50.9)		
Female	105 (79.5)	27 (20.5)	132 (49.1)		
Age (years)				0.42	0.515
2 – 3	72 (76.6)	22 (23.4)	94 (34.9)		
4 – 6	140 (80.0)	35 (20.0)	175 (65.1)		
Birth weight (kg)				3.91	0.048*
Low (< 2.5)	38 (69.1)	17 (30.9)	55 (20.4)		
Normal (≥ 2.5)	174 (81.3)	40 (18.7)	214 (79.6)		
Birth order				0.73	0.393
< 4	164 (80.0)	41 (20.0)	205 (76.2)		
≥ 4	48 (75.0)	16 (25.0)	64 (23.8)		
Parents					
Age (years)					
Father				0.76	0.385
17 – 34	109 (76.8)	33 (23.2)	142 (54.8)		
35 – 56	95 (81.2)	22 (18.8)	117 (45.2)		
Mother				2.22	0.137
17 – 34	146 (76.4)	45 (23.6)	191 (71.0)		
35 – 50	66 (84.6)	12 (15.4)	78 (29.0)		
Educational levels					
Father				10.04	0.018*
No education	23 (69.7)	10 (30.3)	33 (12.7)		
Primary education	93 (73.2)	34 (26.8)	127 (49.0)		
Lower secondary education	37 (88.1)	5 (11.9)	42 (16.2)		
Upper secondary / higher education	51 (89.5)	6 (10.5)	57 (22.0)		
Mother				2.29	0.514
No education	25 (73.5)	9 (26.5)	34 (12.6)		
Primary education	86 (76.1)	27 (23.9)	113 (42.0)		
Lower secondary education	42 (84.0)	8 (16.0)	50 (18.6)		
Upper secondary / higher education	59 (81.9)	13 (18.1)	72 (26.8)		
Employment status					
Father				Fisher exact test	0.347
Working	198 (78.3)	55 (21.7)	253 (97.7)		
Not working	6 (100)	0 (0)	6 (2.3)		
Mother				0.02	0.889
Working	83 (78.3)	23 (21.7)	106 (39.6)		
Not working	128 (79.0)	34 (21.0)	162 (60.4)		
Number of family members				0.001	0.985
< 6	115 (78.8)	31 (21.2)	146 (54.3)		
≥ 6	97 (78.9)	26 (21.1)	123 (45.7)		
Monthly household income (MYR)					
< 1000	138 (76.7)	42 (23.3)	180 (66.9)	1.50	0.221
≥ 1000	74 (83.1)	15 (16.9)	89 (33.1)		

Table 2 shows that 27.7%, 35.2%, and 6.1% of the OA children were underweight, stunted, and wasted, respectively. In contrast, 5.7% were overweight. No significant associations were found between WAZ and BAZ with anemia status of the OA children (p>0.05). On the other hand, stunting was significantly associated with anemia status (χ^2^=7.80, p=0.005), whereby a higher percentage of stunted children (31.2%) were anemic as compared to non-stunted children (16.4%). Furthermore, dietary diversity was not significantly associated with anemia status (p>0.05). On the contrary, mothers' anemia status was significantly associated with the anemia status of their children (χ^2^=13.01, p<0.001), whereby more mothers with anemia (33.0%) had children who suffered from anemia compared to non-anemic mothers (14.3%). The majority of the mothers had abdominal obesity (58.9%) and were overweight or obese (61.8%). However, these factors were not associated with anemia status of the children (p>0.05).

**Table 2:** Nutritional factors of the children and mothers (n = 269). *significant at p<0.05

Characteristics	n (%)			χ^2^	p-value
	Non-anemic (n = 212)	Anemic (n = 57)	Total (n = 269)		
Children					
Weight-for-age (WAZ)				0.73	0.695
Underweight	56 (76.7)	17 (23.3)	73 (27.7)		
Normal	138 (78.4)	38 (21.6)	176 (66.7)		
Overweight/Obesity	13 (86.7)	2 (13.3)	15 (5.7)		
Height-for-age (HAZ)				7.80	0.005*
Non-stunted	143 (83.6)	28 (16.4)	171 (64.8)		
Stunted	64 (68.8)	29 (31.2)	93 (35.2)		
BMI-for-age (BAZ)				0.49	0.782
Wasted	13 (81.3)	3 (18.7)	16 (6.1)		
Normal	174 (77.7)	50 (22.3)	224 (84.8)		
Overweight/Obesity	20 (83.3)	4 (16.7)	24 (9.1)		
Dietary diversity				2.81	0.245
Low	74 (77.9)	21 (22.1)	95 (35.3)		
Medium	110 (76.9)	33 (23.1)	143 (53.2)		
High	28 (90.3)	3 (9.7)	31 (11.5)		
Mothers					
Hemoglobin level (g/dL)				13.01	<0.001*
Non-anemic (≥ 12.0)	138 (85.7)	23 (14.3)	161 (61.0)		
Anemic (< 12.0)	69 (67.0)	34 (33.0)	103 (39.0)		
Waist circumference (cm)				1.61	0.204
Normal (< 80)	85 (83.3)	17 (16.7)	102 (41.1)		
Abdominal obesity (≥ 80)	112 (76.7)	34 (23.3)	146 (58.9)		
Height (cm)				Fisher exact test	1.000
Normal (≥ 145)	190 (78.2)	53 (21.8)	243 (92.0)		
Short stature (< 145)	17 (81.0)	4 (19.0)	21 (8.0)		
Body mass index (kg/m^2^)				4.44	0.109
Underweight (< 18.5)	15 (100)	0 (0)	15 (5.7)		
Normal (18.5–24.9)	65 (76.5)	20 (23.5)	85 (32.4)		
Overweight (25.0–29.9)/Obesity (≥ 30.0)	125 (77.2)	37 (22.8)	162 (61.8)		

Table 3 shows that parasitic infection was significantly associated with anemia status of the children (χ^2^=9.31, p=0.002). A higher percentage of OA children who were infected by intestinal parasites (31.5%) were reported to be anemic as compared to those who were not infected by intestinal parasites (15.2%). Besides, the cleanliness status of the external part of the house was associated with the child’s anemia status (χ^2^=5.35, p=0.021), whereby a higher percentage of children with unclean exteriors of the house (28.9%) were reported to have anemia as compared to their counterparts (16.9%). Similarly, a significantly greater proportion of OA children who did not wear shoes outside the house (42.9%) were anemic as compared to those who did wear shoes outside the house (14.6%; χ^2^=23.13, p<0.001). Furthermore, significantly more children who did not keep their nails clean (28.1%) were anemic as compared to those who maintained nail cleanliness (17.3%; χ^2^=4.30, p=0.038). In general, a high proportion of the children (96.3%) suffered from food insecurity, with more than half of the respondents reporting experiencing child hunger (53.2%), followed by household food insecurity (30.9%) and individual food insecurity (12.3%).

**Table 3:** Household environmental factors of the children (n = 269). *significant at p<0.05

Characteristics	n (%)			χ^2^	p-value
	Non-anemic (n = 212)	Anemic (n = 57)	Total (n = 269)		
Parasitic infection				9.31	0.002*
Negative	140 (84.8)	25 (15.2)	165 (65.0)		
Positive	61 (68.5)	28 (31.5)	89 (35.0)		
Sanitation and hygiene					
Electricity used				0.21	0.647
Yes	187 (79.2)	49 (20.8)	236 (87.7)		
No	25 (75.8)	8 (24.2)	33 (12.3)		
Domestic animal status				2.74	0.098
Yes	165 (76.7)	50 (23.3)	215 (79.9)		
No	47 (87.0)	7 (13.0)	54 (20.1)		
Drink source				4.08	0.130
Pipe water	142 (82.6)	30 (17.4)	172 (63.9)		
Public pipe water/share	14 (70.0)	6 (30.0)	20 (7.5)		
Natural water	56 (72.7)	21 (27.3)	77 (28.6)		
Toilet share status				0.44	0.507
Yes	40 (75.5)	13 (24.5)	53 (19.7)		
No	172 (79.6)	44 (20.4)	216 (80.3)		
Cleanliness status of the house (internal part)				1.46	0.227
Yes	141 (81.0)	33 (19.0)	174 (64.7)		
No	71 (74.7)	24 (25.3)	95 (35.3)		
Cleanliness status of the house (external part)				5.35	0.021*
Yes	143 (83.1)	29 (16.9)	172 (63.9)		
No	69 (71.1)	28 (28.9)	97 (36.1)		
Wash hands before eating				Fisher exact test	0.112
Yes	209 (79.5)	54 (20.5)	263 (97.8)		
No	3 (50.0)	3 (50.0)	6 (2.2)		
Nail cleanliness				4.30	0.038*
Yes	143 (82.7)	30 (17.3)	173 (64.3)		
No	69 (71.9)	27 (28.1)	96 (35.7)		
Wear shoes outside the house				23.13	<0.001*
Yes	176 (85.4)	30 (14.6)	206 (76.6)		
No	36 (57.1)	27 (42.9)	63 (23.4)		
Wash hands after using a toilet				2.36	0.124
Yes	169 (80.9)	40 (19.1)	209 (77.7)		
No	43 (71.7)	17 (28.3)	60 (22.3)		
Wash hands using soap				1.27	0.261
Yes	164 (80.4)	40 (19.6)	204 (75.8)		
No	48 (73.8)	17 (26.2)	65 (24.2)		
Food security				Fisher exact test	1.000
Food secure	8 (80.0)	2 (20.0)	10 (3.7)		
Food insecure	204 (78.8)	55 (21.2)	259 (96.3)		

A total of 18 variables with p<0.25 including birth weight, age of the mothers, father’s education level, monthly household income, child’s HAZ, dietary diversity, mother’s anemia status, waist circumference, BMI, presence of parasitic infection, domestic animal status, drink source, cleanliness status of the house (internal and external parts), nail cleanliness, shoe-wearing outside the house, as well as hand washing before eating and after using a toilet were included in the multiple logistic regression model. Table 4 shows that parasitic infection (adjusted OR (AOR) = 2.49, 95%CI = 1.23-5.06), not wearing shoes outside the house (AOR = 2.95, 95%CI = 1.39-6.27), and mothers with anemia (AOR = 2.62, 95%CI = 1.30-5.28) were significantly associated with higher odds of anemia among OA children.

**Table 4:** Multiple logistic regression of factors associated with anemia. ꭓ^2^ = 28.24, p<0.001. Cox and Snell R^2 ^= 0.116, Nagelkerke R^2 ^= 0.183 *significant at p<0.05

Characteristics	Multiple logistic regression	
	Adjusted OR (95% CI)	p-value
Child’s parasitic infection		
Positive	2.49 (1.23-5.06)	0.011*
Negative	Reference	
Wear shoes outside the house		
Yes	Reference	
No	2.95 (1.39-6.27)	0.005*
Mother’s hemoglobin level		
Non-anemic	Reference	
Anemic	2.62 (1.30-5.28)	0.007*

## Discussion

This study showed a high prevalence of anemia (21.2%) among OA children aged two to six years in Negeri Sembilan, Malaysia. The prevalence was comparable to previous studies conducted among indigenous Temiar OA children under five years old in Kelantan, Malaysia (25.9%) [8] and among Australian Indigenous children aged 24-59 months (22.4%) [21]. Besides, the prevalence of anemia in this study was also comparable to the general Malaysian children under five years old (24.6%) [22]. All these findings highlight the importance of interventions to address childhood anemia.

Parasitic infection was a significant predictor of anemia among OA children in this study. Children infected with parasites were 2.5 times more likely to suffer from anemia. This finding concurred with a previous local study that reported significant associations between *T. trichiura* infection (OR = 2.15, 95%CI = 1.21-3.81) and *A. lumbricoides* infection (OR = 1.63, 95%CI = 1.04-2.55) with iron deficiency anemia [23]. Similarly, the findings of this study are also parallel with a study among children aged 6-59 months in Southern Ethiopia, in which children with parasitic infection were over three times more likely to suffer from anemia (AOR=3.19, 95%CI=1.97-5.17) [24]. The onset of anemia is likely due to the impaired iron re-absorption caused by the ingestion of iron by parasites [23]. Besides, parasites such as *A. lumbricoides* may also impair iron status by sucking blood and damaging the intestinal wall, leading to blood loss and subsequently anemia [25].

A previous local study pinpointed environmental sanitation as an important risk factor for soil-transmitted helminth (*T. trichiura, A. lumbricoides*, and hookworm) infections among OA in Peninsular Malaysia [26]. The current study found that OA children who did not wear shoes outside the house were almost three times more likely to have anemia. A possible explanation is that poor hygiene practices increased their exposure to parasites, consequently leading to anemia. Besides, the lack of shoe-wearing outside the house may be due to their poverty status, which limits the consistent use of shoes and the inability to have frequent shoe replacements [27]. Poverty also reflects a lack of access to nutritious food which may further aggravate anemia [28].

Children whose mothers had anemia were more likely to have anemia. This result was in line with a study among children aged 6-59 months in sub-Saharan Africa, in which mothers with different levels of anemia were associated with a 1.54-2.81 times increased risk of their children having anemia [29]. This is most likely attributed to the similar socioeconomic environment shared by the mothers and their children. These similar exposures may make their children vulnerable to anemia. For example, mothers and their children with similar exposure to infections such as malaria may have compromised production of red blood cells and iron stores, and subsequently, end up with anemia [30].

There are several limitations in this study. Firstly, the cross-sectional study design was unable to establish a causal relationship between the risk factors and the onset of anemia among the study population. Cohort studies are warranted to support and confirm the determinants of anemia among OA children. Secondly, the findings of this study cannot be generalized to all OA children in Peninsular Malaysia as it only focused on two districts in the state of Negeri Sembilan. Future studies should be conducted in each state in Peninsular Malaysia to obtain a representative study population. Furthermore, hemoglobin concentration was used as a proxy indicator of anemia in this study, and the full panel of serum ferritin, iron, and vitamin B12 was not analyzed. Future studies should include serum ferritin, iron, and vitamin B12 to identify the different types of anemia. Other inflammatory parameters such as interleukin-6 (IL-6) and C-reactive protein (CRP) should also be included to provide a more comprehensive picture of the anemia status of the OA children. Lastly, further studies should look into the roles of other factors such as chronic illness, family history of different types of anemia, breastfeeding practices, and iron supplementation during pregnancy in relation to the anemia status of OA children.

Despite these limitations, the findings from this study explored the associations between nutritional factors and household environmental factors with the anemia status of OA children aged two to six years in Malaysia, which have not been previously reported. This study adds to the body of knowledge on the anemia status of preschoolers in a local context. Increased knowledge of the potential predictors of anemia status is beneficial for stakeholders as they will be able to develop intervention programs to improve socioeconomic status as well as the nutritional and health status of the OA population, thus reducing anemia.

## Conclusions

Anemia status among OA children remains an important public health concern as evidenced by the high prevalence rate of anemia in this study. The findings of this study indicated that OA children who did not wear shoes outside their house, had parasitic infections, and with mothers with anemia were significantly associated with anemia. This study highlighted that adequate nutritional status and good sanitation and hygiene status play important roles in determining the hemoglobin level of OA children. Therefore, periodic deworming of OA children together with the improvement in personal hygiene and sanitation would be important in the public health strategies for the prevention of anemia among OA children.
